# Lord Derby to Dr. Denman

**Published:** 1800-07

**Authors:** 

**Affiliations:** Grosvenor Square


### Lord DERBY to Dr. DENMAN.

Dear Sir,

I have great pleaſure in ſending you as exact an account as I can give, of a circumſtance which occurred when my two children were inoculated for the Cow-pox; and if you think the knowledge of it may prevent any dangerous conſequences to others, you have my permiſſion to make it public.

April 30,—the children, one of which was fourteen months and the other two months old, were inoculated for the Cow-pox, by Mr. Knight, from the arm of a child which had alſo been inoculated by him. On the next morning, the arm of the elder child was much inflamed, and in a few hours a ſmall veſicle appeared on the punctured part, which ſo nearly reſembled the appearance we had been taught to expect, that we concluded ſhe had received the infection. When Mr. Knight viſited the child, he expreſſed many doubts, becauſe, as I underſtood, the inflammation ſo ſpeedily followed the operation. Theſe doubts were ſoon converted into certainty, as in a ſhort time the inflammation ſubſided, and a ſlight incruſtation was formed on the veſicle, which he aſſured me had not the true character of the Cow-pox; though I was, and many others not converſant with the diſeaſe might have been, miſled by the appearance. On the Monday following, ſhe was inoculated in both arms, with matter taken from a different ſubject; and this was followed by a ſucceſſion of preciſely the ſame appearances as thoſe above deſcribed. On Wedneſday, ſhe was again inoculated from the arm of her brother, in whom the diſeaſe had gone on ſatisfactorily from the firſt inoculation. This third inoculation ſucceeded completely, the arm obſerving the ſame courſe as that of her brother. Neither of the children had the ſmalleſt appearance of fever or indiſpoſition during the whole proceſs. I am,

With great regard, &c.

